# Supplementary material for: 5-Fluorouracil Encapsulated Chitosan-Cellulose Fiber Bionanocomposites: Synthesis, Characterization and In Vitro Analysis towards Colorectal Cancer Cells
Source: Nanomaterials (Basel). 2021 Jun 28;11(7):1691. doi: 10.3390/nano11071691 (PMC8305564; doi:10.3390/nano11071691)
Supplement: Supplementary file 1 [file nanomaterials-11-01691-s001.zip › nanomaterials-1191250-supplementary.pdf]

nanomaterials

Supplementary data

5-Fluorouracil Encapsulated Chitosan-Cellulose Fiber Bionanocomposites: Synthesis, Characterization and In Vitro Analysis towards Colorectal Cancer Cells

Mostafa Yusefi [1], Hui-Yin Chan [2], Sin-Yeang Teow [2], Pooneh Kia [3], Michiele Lee-Kiun Soon [2], Nor Azwadi Bin Che Sidik [1,*] and Kamyar Shameli [1,*]

1 Malaysia-Japan International Institute of Technology, Universiti Teknologi Malaysia, Jalan Sultan Yahya Petra, 54100, Kuala Lumpur, Malaysia; myusefi175@gmail.com (M.Y.)

2 Department of Medical Sciences, School of Medical and Life Sciences, Sunway University, Jalan Universiti, Bandar Sunway, 47500 Selangor Darul Ehsan, Malaysia; sin-yeang ronaldt@sunway.edu.my (S-Y.T.); 19117936@imail.sunway.edu.my (M.L-K.S.)

2 Institute of Bio Science, University Putra Malaysia, 43400 UPM Serdang, Selangor, Malaysia, kia.pooneh@gmail.com (P.K.)

* Correspondence: kamyarshameli@gmail.com; azwadi@utm.my

Citation: Yusefi, M.; Chan, H.-Y.; Teow, S.-Y.; Kia, P.; Soon, M.L.-K.; Sidik, N.A.B.C.; Shameli, K. 5-Fluorouracil Encapsulated Chitosan-Cellulose Fiber Bionanocomposites: Synthesis, Characterization and In Vitro Analysis towards Colorectal Cancer Cells. *Nanomaterials* **2021**, *11*, 1691. https://doi.org/10.3390/nano11071691

Academic Editor: Carla Vilela, Carmen S. R. Freire and Eleonore Fröhlich

Received: 4 April 2021
Accepted: 19 May 2021
Published: 28 June 2021

Publisher's Note: MDPI stays neutral with regard to jurisdictional claims in published maps and institutional affiliations.

Copyright: © 2021 by the authors. Licensee MDPI, Basel, Switzerland. This article is an open access article distributed under the terms and conditions of the Creative Commons Attribution (CC BY) license (http://creativecommons.org/licenses/by/4.0/).

2. Materials and Methods

2.3.6. Antibacterial Assay

A broth micro-dilution method was used to determine the minimum inhibitory concentration (MIC) of the samples using the Clinical and Laboratory Standards Institute (CLSI) protocols as described previously [35,36]. Gram-positive (*Staphylococcus aureus* ATCC 23235) and Gram-negative (*Escherichia coli* ATCC 11775) bacterial strains were used in this study. Single colony of fresh bacterial culture (12–18 h) was isolated from Mueller Hinton agar (MHA) plates and inoculated into sterile Mueller Hinton broth (MHB). The culture was grown overnight (12–18 h) prior to the experiments. Next day, the bacterial concentration was standardized to an optical density (OD) of 600 nm (approximately 10^8 CFU/mL) with MHB. Two-fold serial dilutions of samples were prepared in 96-well plates to give final test concentrations of 0, 15.62, 31.25, 62.5, 125, 250, 500 and 1000 µg/mL per well. 10 µL of bacterial suspension equivalent to 10^6 CFU/mL of exponentially growing bacterial cells were added to the wells. The plates were incubated at 35 ± 2 °C for 18 h. Following the overnight incubation, the plate was then read for the absorbance at 600 nm using microplate reader (Tecan) to determine the MIC values. Three independent experiments were performed and the data are expressed as the mean ± standard deviation for all triplicates within an individual experiment.

3. Results and Discussion

3.10. Antibacterial Assay

As CS, CS-based nanoparticles and nanocomposites have been known with their potent antibacterial action against a broad range of bacterial strains [1,2]. We tested the antibacterial potential of CS-containing compounds along with CF. However, no significant antibacterial activity of CF, CS NPs, CS-CF BNCs and CS-CF/5-FU BNCs was seen in Gram-positive *Staphylococcus aureus* and Gram-negative *Escherichia coli*.

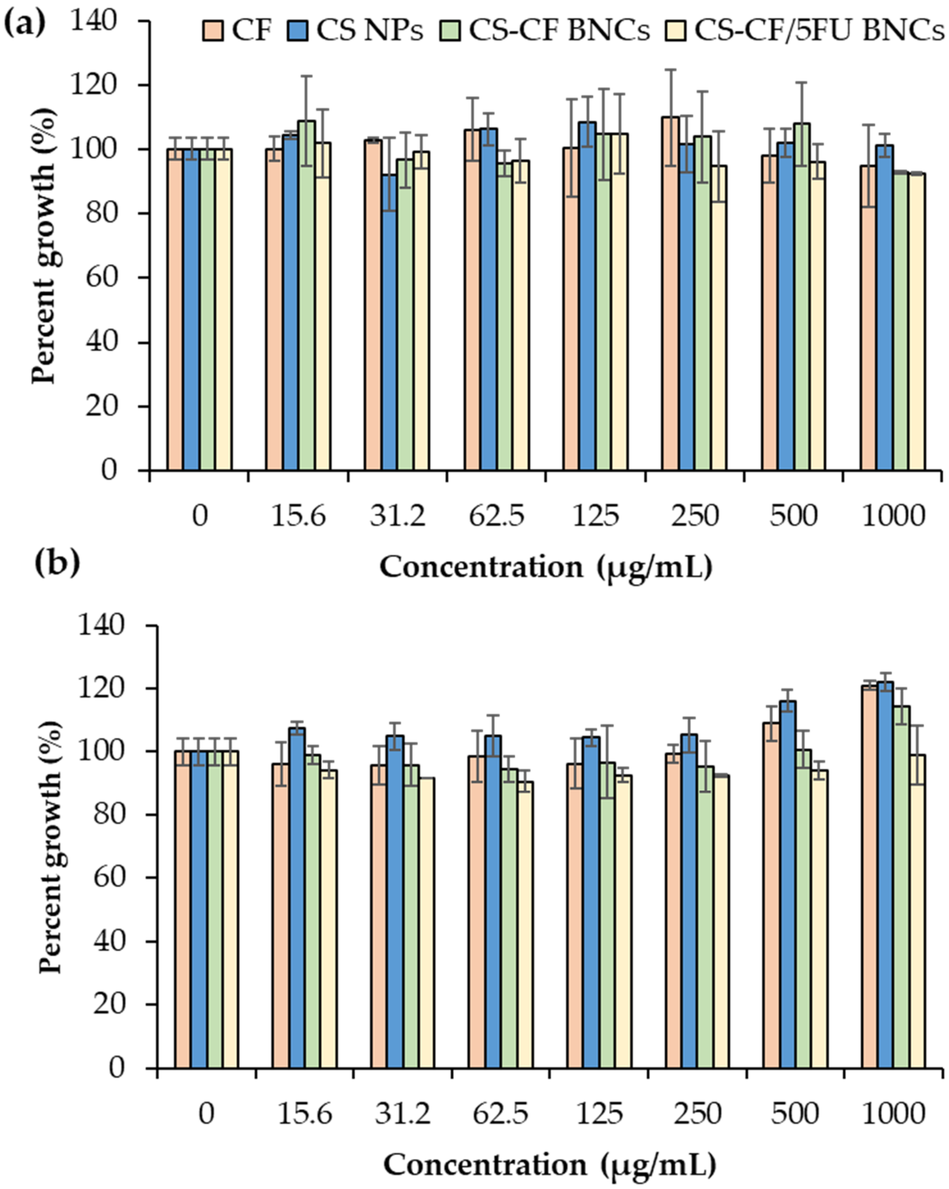

Figure S1. Antibacterial activity of CF, CS NPs, CS-CF BNCs and CS-CF/5-FU BNCs against (a) Gram-positive *Staphylococcus aureus* and (b) Gram-negative *Escherichia coli.*

References

1. Chandrasekaran, M.; Kim, K.D.; Chun, S.C. Antibacterial Activity of Chitosan Nanoparticles: A Review. *Processes* **2020**, *8*, 1173.
2. Kravanja, G.; Primožič, M.; Knez, Ž.; Leitgeb, M. Chitosan-based (Nano) materials for novel biomedical applications. *Molecules* **2019**, *24*, 1960.
